# Conservation analysis and pathogenicity prediction of mutant genes of ectodysplasin a

**DOI:** 10.1186/s12881-018-0726-2

**Published:** 2018-12-07

**Authors:** Fangqi He, Hongfeng Wang, Xiaoyu Zhang, Qingping Gao, Feng Guo, Chang Chen

**Affiliations:** 1 Department of Prosthodontics, Xiangya Hospital, Central South University, Changsha, 410008 Hunan China; 2 Department of Prosthodontics, Changsha Stomatological Hospital, Changsha, 410004 Hunan China

**Keywords:** Hypohidrotic ectodermal dysplasia, Ectodysplasin a gene, Gene mutation, Related homologous proteins, Conservation, Pathogenicity

## Abstract

**Background:**

Hypohidrotic ectodermal dysplasia (HED) is a common recessive X-linked hereditary disease that affects the development of ectoderm. Gene mutations of ectodysplasin A (EDA) play key roles in process of this disease. In our preliminary study, three unknown mutation sites (c.878 T > G, c.663-697del and c.587-615del) were detected from the pedigrees of HED.

**Methods:**

Conservation analysis of the related homologous proteins in 3 unknown EDA gene mutation sites was conducted using the University of California Santa Cruz (UCSC) Genome Browser database. SIFT and PolyPhen-2, the online gene function prediction software, were utilized to predict the pathogenicity of point mutation of c.878 T > G.

**Results:**

All three unknown mutation sites were located in the highly-conserved region of EDA and possessed strong amino acid conservation among different species. In addition, the results of the pathogenicity prediction of point mutation of c.878 T > G by SIFT (*P* = 0.00) and PolyPhen-2 (S = 0.997) demonstrated that the mutation site had considerable pathogenicity theoretically.

**Conclusions:**

The EDA mutations of c.878 T > G, c.663-697del and c.587-615del may be responsible for the pathogenesis of HED in their pedigrees.

## Background

Hypohidrotic ectodermal dysplasia (HED), also known as congenital ectodermal dysplasia syndrome, is a group of common hereditary diseases that affect the development of ectoderm. It often affects the development of more than two ectoderm-derived organs. The prevalence is approximately 1/100000 [1]. Clinical manifestations of HED mainly include hypotrichosis, loss or maldevelopment of sweat glands and nail dysplasia [2]. The main oral symptoms are alveolar bone dysplasia, craniofacial deformity and loss of intermaxillary distance [3, 4]. A majority of patients present with congenital tooth dysplasia, small incisors or canine teeth in cone shape and even full-mouth anodontia [5, 6].

Most HED cases have an X recessive inheritance [7, 8] and their pathogenic gene is the EDA gene [9]. Approximately 95% of the total HED patients are induced by gene mutations such as nonsense mutation, missense mutation and small fragment deletion [10]. Like most recessive X-linked hereditary diseases, the HED pedigree members are primarily male, whereas the female carriers merely present with mild clinical manifestations [11]. About 30% of the female carriers are asymptomatic [12].

In a preliminary study carried out by authors of the current study, peripheral blood of 4 unrelated HED pedigrees was collected. The peripheral blood of 100 healthy adults who don’t have the family history of congenitally missing tooth was used for comparison. The 4 symptomatic patients were subjected to a thorough clinical examination. Four male probands had typical clinical manifestations which were in line with X-linked recessive inheritance. The gene sequencing results demonstrated that the mutation was only found in the symptomatic patients and had patient to patient variability [13], including two point mutations (c.466C > T, c.878 T > G) and two deletion mutations (c.663-697del, C.587-615del). When compared with the reported gene mutation sites, point mutation (c.466C > T) in one pedigree was identified as a pathogenic mutation. The same is reported by Monreal 1988 [14]. The other three mutation sites (c.878 T > G, c.663-697del and c.587-615del) were new unidentified mutations.

SNP (single-nucleotide polymorphism) is the most common genome mutation. There are about a million SNP loci among humans. The SNP that is located in the gene coding region and can change the amino acid sequence leading to a missense mutation. With the improvement in disease diagnosis, there is a higher possibility of discovering new missense mutations [15]. In current times, researchers tend to use the different kinds of bioinformatics software to make the preliminary forecasting of the pathogenicity of the new mutation, recognize the related risks of the disease and offer the theoretical foundation using the cell model and the mouse disease model. In this study, conservation analysis of the related homologous proteins in 3 unknown EDA gene mutation sites was conducted by referring to the UCSC Genome Browser database. The online gene function prediction software SIFT and PolyPhen-2 were adopted to predict the pathogenicity of point mutation of c.878 *T*>. The aim of the study is to offer a reference for elucidating the pathogenesis of HED.

## Methods

### Database selection

Human Genome version 19 (hg19), the reference version of the human genome in the UCSC database, was used as the reference genome data for conservative analysis of the sequence alignment and related homologous protein of mutation sites of c.878 T > G, c.663-697del and c.587-615del in EDA gene among different species. These species included human, chimpanzee, gorilla, rhesus, mouse, rat, dolphin, horse, dog, chicken and zebrafish.

### Online gene function prediction

SIFT and Polyphen-2 are the two most commonly used online gene function prediction software. Their parameters are the system default. The above were used to make the function prediction of the SNP and the point mutation.

The software SIFT(Sorting Intolerant From Tolerant)(http://sift.jcvi.org/), based on the prediction principle of protein sequence homology-based tool, predicts the influence of amino acid substitution. The predict outcomes of amino acid substitutions that are caused by nucleotide mutation is calculated as a standardized score whose range of variation ranges from 0 to 1. There are four criteria for rating: tolerated low confidence, tolerated, deleterious low confidence and deleterious. When the score of SNP is less than 0.5, it means the mutation is deleterious. Hence, the SNP has a great influence on protein function. Therefore, a lower SNP score implies increasing harm.

The software syphen2 (Phenotyping Version2)(http://genetics.bwh.harvard.edu/pph2/), based on the prediction principle of protein structural homology-based method, adopts the machine learning algorithm of Naive Bayes to assess the stability influence caused by amino acid change of SNP on the folding, interaction and conformation of the protein. In the predict outcomes, the SNP is also assigned a score. The difference from SIFT is that, an increase in the SNP score implies increasing harm. There are four criteria for rating: unknown, benign, possibly damaging and probably damaging. When the score is larger than 0.9, it means the mutation is probably damaging.

## Results

### Gene detection in HED pedigrees

**Fig. 1:**
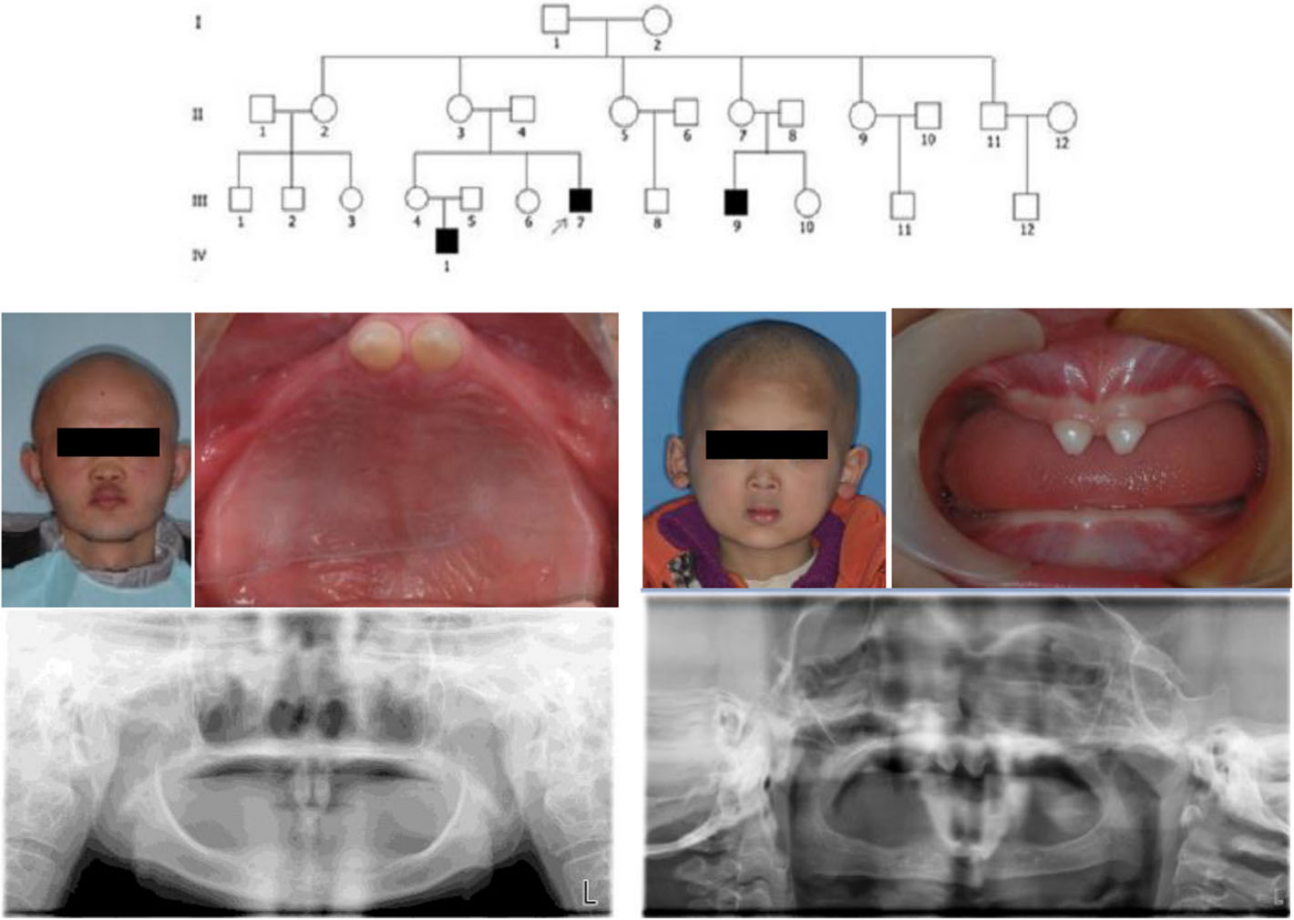
The family tree of C.878 T > G gene mutation patients affected by HED (upper) and their phenotypic appearance of the proband (lower left) and his nephew (lower right). Notes: HED, hypohidrotic ectodermal dysplasia

In our preliminary investigation, 4 unrelated HED pedigrees were chosen for genetic testing and 3 unknown EDA mutations were detected. These included c.878 T > G, c.663-697del and c.587-615del. Drawing from previous experiments, conservation analysis of relevant homologous proteins in three unknown mutations of EDA gene was performed in reference to the UCSC Genome Browser database. The pedigrees of probands (c.878 T > G) belonged to a large clinical pedigree. The probands (III7), his nephew (IV1) and younger male cousin (III9) presented with identical manifestations. The results of gene detection showed that there were c.878 T > G missense mutation in the EDA gene in the results of the propositus, his nephew and younger male cousin. There is heterozygous mutation c. 878 T > G in the EDA gene of carriers (I2,II9,III4). No mutation was found in normal subjects (II2,II3,II11,III6). The above is the genotype and phenotype co-segregation phenomenon. It can be speculated that this mutation is a pathogenic mutation from the gene level (Fig. 1).

### Conservation analysis of gene mutations of EDA gene

**Fig. 2:**
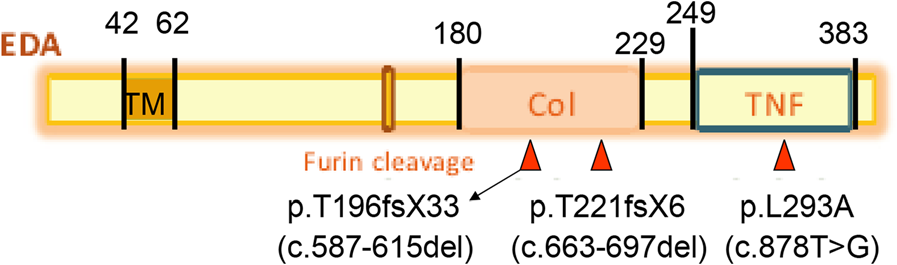
The locations of three unknown EDA gene mutation sites in the functional domain of the EDA gene. Notes: EDA, ectodysplasin A; TM, transmembrane domain; Furin cleavage, furin enzyme binding site; Col, collagen-like domain TNF

**Fig. 3:**
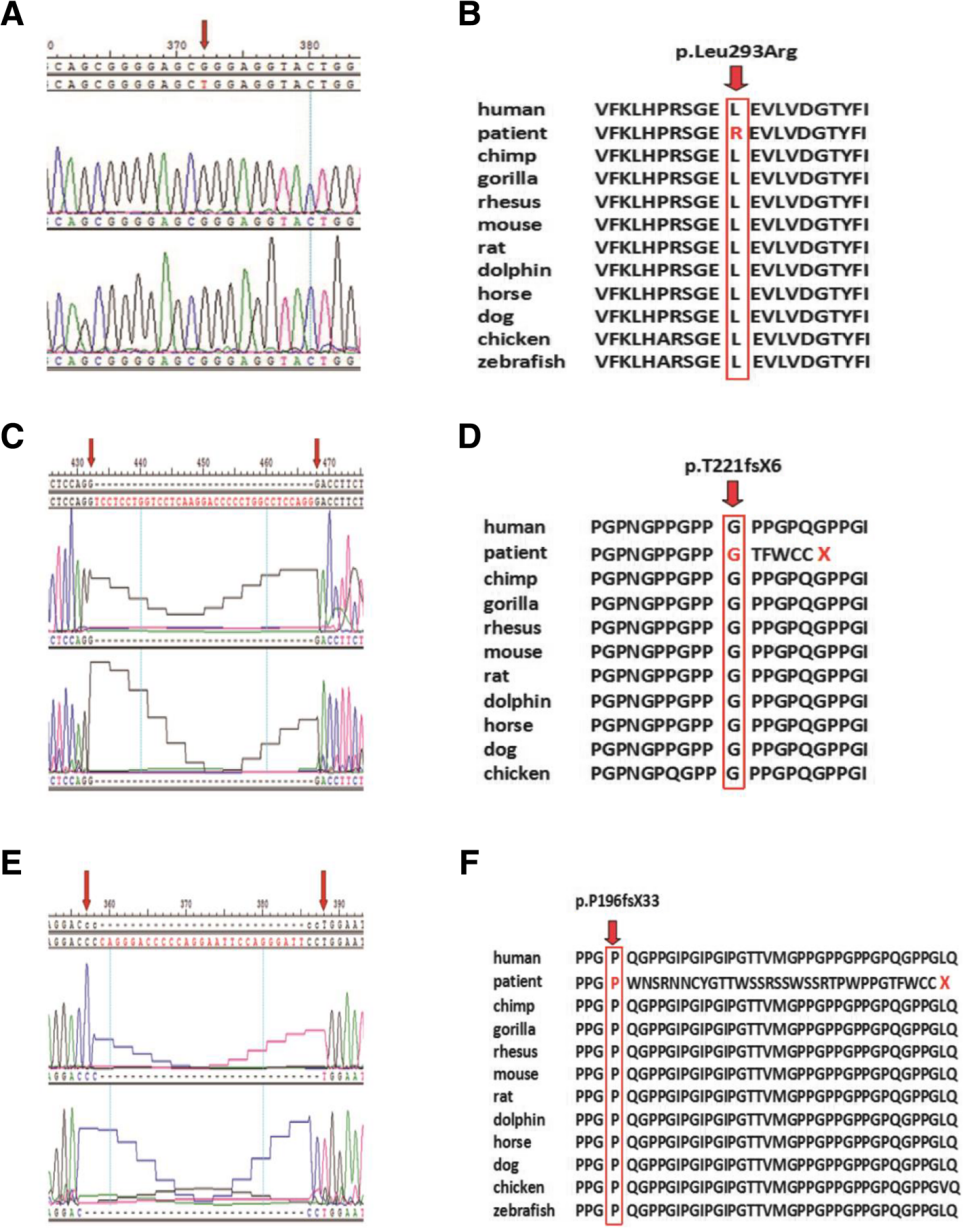
Comparing the conservatism of three unknown EDA gene mutation sites among different species. (**a**) EDA gene mutation peak in the pedigree of c.878 T > G; (**b**) Conservation analysis of c.878 T > G mutation site among different species; (**c**) EDA gene mutation peak in the pedigree of c.663-697del; (**d**) Conservation analysis of c.663-697del mutation site among different species; (**e**) EDA gene mutation peak in the pedigree of c.587-615del; (**f**) Conservation analysis of c.587-615del mutation site among different species. These species included human, chimpanzee, gorilla, rhesus, mouse, rat, dolphin, horse, dog, chicken and zebrafish

The mutation sites of EDA gene including p.L293R (c.878 T > G), p.T221fsX6 (c.663-697del) and p.P196fsX33 (c.587-615del) possessed strong amino acid conservation among different species, prompting that these three newly-discovered mutation sites of EDA gene were probably located in the vital function region of the coding genes during the process of species evolution (Fig. 2, Fig. 3). These findings were consistent with the genetic testing results of three unknown mutation sites.

### Pathogenicity prediction of mutation site of c.878 T > G in EDA gene

**Fig. 4:**
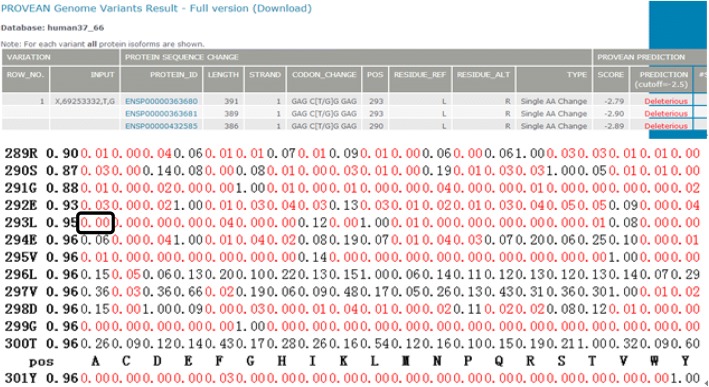
The pathogenicity prediction of the mutation site of c.878 T > G using the software SIFT. The value of L293A was *P* = 0.00, and a *P* value < 0.05 was considered to probably affect the protein function

**Fig. 5:**
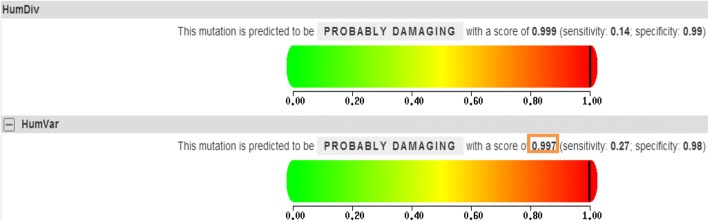
The pathogenicity prediction of the mutation site of c.878 T > G using the software PolyPhen-2. The mutation prediction is based on the results of second graph HumVar. The closer the score is to 0.00, the less possibility causing protein damage. The score of c.878 T > G was 0.997, which was close to 1.0, indicating that it theoretically had high pathogenicity and potentially caused damages

The pathogenicity of the mutation site of c.878 T > G was evaluated. SIFT revealed that the score of L293A was 0.00 and a score of < 0.05 was considered to probably affect the protein function (Fig. 4*)*. PolyPhen-2 demonstrated that the pathogenicity of mutation site of c.878 T > G was 0.997, which was close to 1.0, indicating that it theoretically had high pathogenicity and potentially caused damages (Fig. 5). The results above implied that this mutation site possessed strong pathogenicity theoretically.

## Discussion

EDA gene is a causative gene of HED, which is located in the Xq12-q13 region. It contains 9 exons and encodes 391 amino acids. The encoded EDA protein is a type II trans-membrane protein with a short intracellular domain at the N-terminal and a TNF homology domain with 19 glycine residual repetitive sequence at the C-terminus [16]. It has multiple subtypes and mainly mediates epidermal-mesenchymal and cell-cell signaling transduction [17]. As an important member of tumor necrosis factor ligand family, EDA protein has been extensively studied in the field of developmental biology. It can regulate body morphogenesis and maintain the development of ectoderm-derived organs (tooth, hair, sweat gland and other cutaneous appendages) [18, 19]. Previous studies have demonstrated that application of the EDA gene-transfected bone mesenchymal stem cells can significantly improve the regenerative capacity of sweat glands and accelerate the potential repair and regeneration of the injured skin and its appendages [20]. However, after the EDA gene-inhibiting antibodies were implanted in wild-type pregnant mice, the wild-type mouse fetuses were found to have permanent ectodermal dysplasia [21].

EDA gene mutations are mainly located in four important functional areas [22]. ① The junction (TM) between the trans-membrane and extra-cellular parts which is related to the polarity of the amino acids. ② Furin cleavage is associated with the production of soluble molecules. The activity can be activated by the release of the soluble form of EDA proteins.③ Collagen-like domain (collagen) can bind two or more EDA trimer together, thereby stimulating the downstream signaling pathway.④ TNF homology domain (THD) can affect the formation of trimer and its specific binding with receptors. More than 100 types of EDA mutations have been found to have heterogeneity and ethnic differences [23, 24]. It is reported that 95% of the gene mutations occur in the 1, 3, 5, 8 and 9 exons, including missense mutation, small fragment insertion or deletion mutation and nonsense mutation [25, 26]. In addition, the deletion or translocation of single exon or full-length gene fragments has also been reported [27].

Mutations in the EDA gene can not only lead to congenital anodontia syndrome, but also provoke congenital anodontia with relatively mild symptoms, which are not complicated with the developmental defects of ectoderm-derived tissues and organs [26]. Tao et al. have reported of a Mongolian family with congenital anodontia. Besides congenital anodontia, no other clinical manifestations of HED were present in the affected family members. A new missense mutation (c.198C > G) was found in the EDA gene among all affected male and female carriers [28]. Song et al. [29] collected 15 unrelated male patients with asymptomatic congenital anodontia. Gene mutation testing revealed three novel mutation sites (p.Ala259Glu, p.Arg289Gys and p. Arg334His) in EDA gene of four patients with a detection rate of 27%. The genetic defects of EDA gene could lead to the incidence of asymptomatic congenital anodontia.

Along with the development of genetics and genomics fields, the EDA genes originally found in both patients and mouse models have been proven to contain a highly-conserved TNF homology domain in vertebrates and mammals [22, 30]. The findings in this study have demonstrated that the c.878 T > G mutation is located in the TNF homology domain of EDA gene. Mutations in this region can affect the binding of EDA protein to its receptors, resulting in the inactivation of NF-κB signaling pathway, thereby leading to the developmental defects of ectoderm-derived tissues and organs. NF-κB signaling pathway is now recognized as a classical signaling pathway associated with the incidence of HED [31]. C.878 T > G family is a large clinical pedigree. The nephews and cousins of the probands shared similar manifestations. Genetic testing showed that the base 878 of exon 7 was mutated from thymine to guanine in EDA gene of the probands and their nephews, and thus the CTG code was mutated into CGG, resulting in a change from leucine to arginine at the amino acid 293 (p.Leu293Arg). Genotype testing of this mutation at the gene level is the cause of hypoplastic ectodermal hypoplasia in this pedigree. In addition, further examination was carried out using the prediction software SIFT and PolyPhen-2. The results indicated that the mutation site possessed strong pathogenicity theoretically. The databases of 1000 Genome Project, the NHLBI GO Exome Sequencing Project and dbSNP database, were used to identify the frequency of the mutations. This further supported the pathogenicity of this mutation. However, the specific mechanism is not well understood. The study for the new missense mutation (c.878 T > G) aims to offer the theoretical support for the next functional experiment. Next, our research group will transfect the eukaryotic cell that is related to tooth development, and make a evaluate the effects of mutated EDA genes on tissues and organs of ectodermal origin at the protein level with a goal of guiding the clinical treatment.

The other two mutations (c.663-697del, c.587-615del), which have not been reported, are located in the collagen-like domain of EDA gene. These mutations lead to the frame-shifting mutation of exon 4 and induce a termination codon in advance at the 226th and 238th amino acids, resulting in a truncated protein. This causes functional defects of the EDA protein, which is unable to form normal EDA trimer, weakens the activation of downstream signaling pathways and affects the development of ectoderm-derived tissues and organs. Due to the complexities involved in simulating computer algorithms, the numerical procedure to predict the functional effects of these two unknown deletion mutations was not used. This may be carried out further studies.

## Conclusions

Three unknown mutation sites (c.878 T > G, c.663-697del and c.587-615del) from the pedigrees of HED possess strong conservation of amino acid among different species and the mutation site of c.878 T > G holds strong pathogenicity theoretically. This may be responsible for the pathogenesis of HED in their pedigrees and it can also provide a reference for the pathogenicity of a wide range prediction of gene mutations.

## Abbreviations

EDA: ectodysplasin A
HED: Hypohidrotic ectodermal dysplasia
UCSC: the University of California Santa Cruz
